# Safety and Pitfalls of Blepharoptosis Surgery in Elderly People

**DOI:** 10.1055/s-0043-1770082

**Published:** 2023-08-28

**Authors:** Yuji Shirakawa, Kazuhisa Uemura, Shinji Kumegawa, Kazuki Ueno, Hiroki Iwanishi, Shizuya Saika, Shinichi Asamura

**Affiliations:** 1 Department of Plastic and Reconstructive Surgery, Wakayama Medical University, Wakayama City, Wakayama, Japan; 2 Department of Ophthalmology, Wakayama Medical University, Wakayama City, Wakayama, Japan

**Keywords:** blepharoptosis, elderly patients, superficial punctate keratopathy, margin reflex distance-1, subjective symptoms

## Abstract

**Background** Elderly patients often have complications of blepharoptosis surgery that can result in the appearance or exacerbation of superficial punctate keratopathy (SPK). However, postoperative changes to SPK status have not been previously reported. We used subjective assessment of symptoms and measurement of SPK scale classification to investigate the safety and efficacy of blepharoptosis surgery in elderly patients.

**Methods** Included in this prospective study were 22 patients (44 eyes) with bilateral blepharoptosis that underwent surgery. Patients comprised 8 males and 14 females with a mean (±standard deviation) age of 75.7 ± 8.2 years (range: 61–89). Blepharoptosis surgery consisted of transcutaneous levator advancement and blepharoplasty including resection of soft tissue (skin, subcutaneous tissue, and the orbicularis oculi muscle). Margin reflex distance-1 (MRD-1) measurement, a questionnaire survey of symptoms and SPK scale classification, was administered preoperatively and 3 months postoperatively for evaluation.

**Results** The median MRD-1 was 1 mm preoperatively and 2.5 mm postoperatively, representing a significant postoperative improvement. SPK area and density scores were found to increase when the MRD-1 increase was more than 2.5 mm with surgery. All 10 items on the questionnaire tended have increased scores after surgery, and significant differences were observed in 7 items (poor visibility, ocular fatigue, heavy eyelid, foreign body sensation, difficulty in focusing, headaches, and stiff shoulders).

**Conclusion** Blepharoptosis surgery was found to be a safe and effective way to maintain the increase in MRD-1 within 2.0 mm. Despite the benefits, surgeons must nonetheless be aware that blepharoptosis surgery is a delicate procedure in elderly people.

## Introduction

Blepharoptosis (BP) surgery is commonly performed in both young and elderly patients with the goal of broadening the visual fields. 1 2 3 4 5 6 The majority of BP surgery is performed for dehiscence or stretching of the aponeurosis to the tarsus. It is achieved by nondelivery of contractile power to the upper eyelid, which requires resection of redundant skin and/or shortening of the levator aponeurosis. 7 8 However, when evaluating the surgical outcomes, most clinical surgeons that perform BP tend to focus on cosmetic improvements, such as upper-eyelid contour and left-right difference in the pretarsal show. 5 9 10

BP surgery widens the visual field and cosmetic improvement can be obtained, but not all patients are satisfied postoperatively. Elderly patients can have functional problems after BP surgery that differ from younger patients, such as visual disturbance and dry eye symptoms. 11 12 Wider discussion of BP surgery is therefore needed, as various subjective symptoms in elderly patients require improvement.

Elderly patients are known to often have complications from BP surgery that can result in the appearance or exacerbation of superficial punctate keratopathy (SPK); however, there are no reported cases of pre- and postoperative changes in SPK status. This study, therefore, aims to investigate the safety and efficacy of BP surgery in elderly patients using subjective symptoms and measurement of SPK scale classification.

## Methods

Included in the study were the 22 patients (44 eyes) with bilateral BP surgery that underwent surgery at our hospital between April 2019 and February 2020. Patients comprised 8 males and 14 females with a mean (±standard deviation) age of 75.7 ± 8.2 years (range: 61–89). Exclusion criteria were inability to answer the questionnaires and the inability to attend the 3-month follow-up visit. We also excluded patients with congenital, neurogenic, myogenic, and traumatic ptosis and those with history of glaucoma. Patients with neurogenic or myogenic ptosis were ruled out after consultation with members of our department of neurology. Informed consent was obtained from each patient before the start of the study. This study was approved by an appropriate institutional review board (approval number 2549), and performed in accordance with the tenets of the Declaration of Helsinki.

BP surgery always consisted of transcutaneous levator advancement, and blepharoplasty including resection of soft tissue (skin, subcutaneous tissue, and the orbicularis oculi muscle). 1 13 14 15 Intraoperative quantification in cases of transcutaneous levator advancement was conducted so that margin reflex distance-1 (MRD-1) was more than or equal to 4.0 mm while in the supine position. MRD-1 measurement, a questionnaire survey of symptoms, SPK grading, and BUT (tear breakup time test) value were taken pre- and 3 months postoperatively for evaluation.

### Margin Reflex Distance-1 Measurement

The upper eyelid height before and after surgery was assessed in primary position using MRD-1 value, deﬁned as the distance between the central pupil reﬂex and the upper eyelid margin. 16

1. Questionnaire survey of symptoms

The degree of disability was scored on a Likert scale ranging from 1 to 5, with lower numbers indicating a greater burden. Patients answered a 10-item questionnaire on eye symptoms including poor visibility, ocular fatigue, heavy eyelids, painful or sore eyes, dry sensation in the eyes, foreign body sensation, and difficulty in focusing and double vision, and it included accompanying symptoms, such as headaches and stiff shoulders.

2. SPK scale

**Fig. 1:**
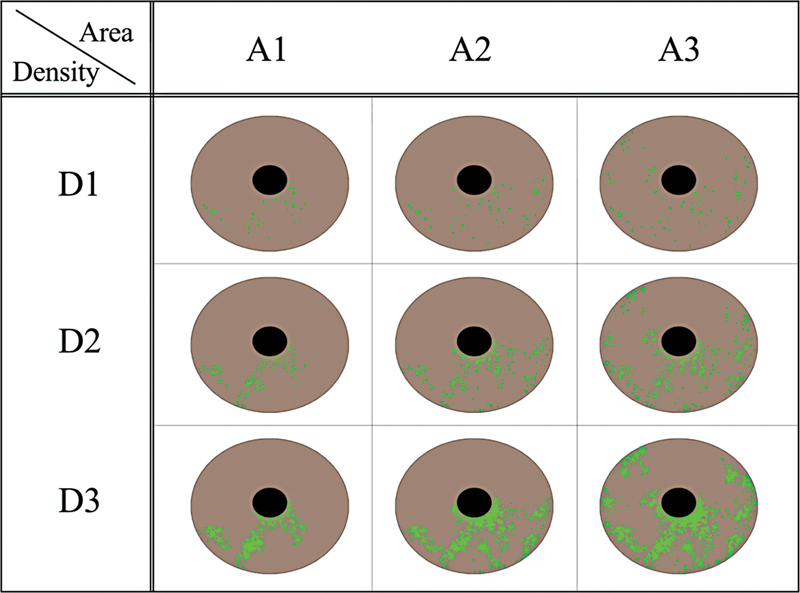
Superficial punctate keratopathy and density scale classification. Area and density were evaluated on a 4-point scale (none: 0, 1: mild, 2: moderate, 3: severe).

The presence of any corneal staining was documented as follows: after administration of fluorescein in the inferior fornix using a paper fluorescein strip (FLUORES Ocular Examination Test Paper 0.7 mg; Ayumi Pharmaceutical, Tokyo, Japan), the corneal epithelium and BUT were examined using a slit-lamp (RO-5000; Rodenstock, Munich, Germany). SPK was graded using the area and density of the lesion as parameters. Area and density were evaluated on a 4-point scale according to the method used by Miyata et al (0: none, 1: mild, 2: moderate, 3: severe). 17 After fluorescein staining, the total sum of the area of SPK was graded as A0 when there was no punctate staining, A1 when the area occupied less than one-third of the cornea, A2 when the area occupied between one- and two-thirds of the cornea, and A3 when the area occupied more than two-thirds of the cornea. The density was graded as D0 when there was no punctate staining, D1 when the density was sparse, D2 when the density was moderate, and D3 when the density was high or the lesions overlapped ( Fig. 1 ). 17 We collaborated with an ophthalmologist regarding measurements and examinations. Since these assessments involve a subjective component, the co-authors, a plastic surgeon (K.U) and an ophthalmologist (H.I), carefully considered each case when determining the SPK scale.

### Statistical Analysis

Continuous variables were analyzed using the Mann–Whitney U test and Wilcoxon signed-rank sum test. A receiver operating characteristic curve was created, and the area under the curve was calculated. A *p* -value less than 0.05 was considered statistically significant. All statistical analyses were done using JMP 14 software (SAS Institute Inc., Cary, NC).

## Results

**Fig. 2:**
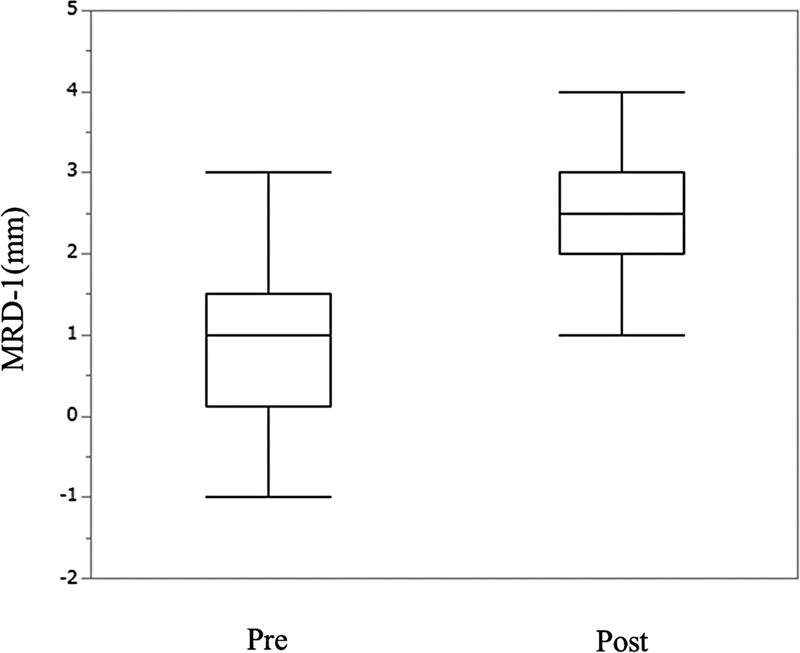
Pre- and postoperative margin reflex distance-1 (MRD-1). The median MRD-1 was 1 mm (interquartile range: 0.125–1.5) preoperatively and 2.5 mm (interquartile range: 2–3) at 3 months postoperatively. Boxes indicate medians; error bars, upper and lower limits.

**Fig. 3:**
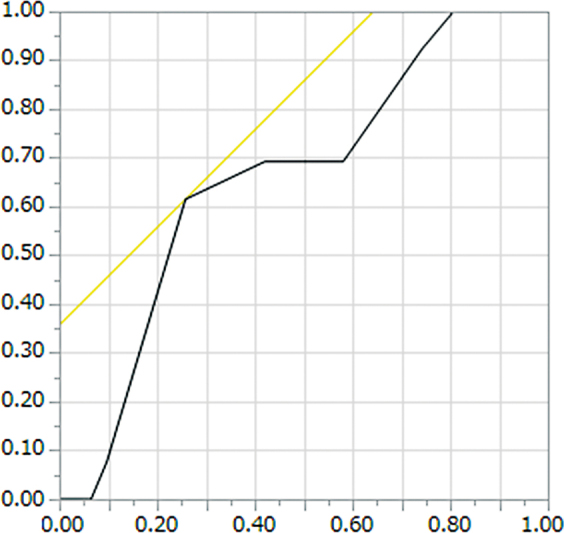
Receiver operating characteristic curve for the support tool to detect margin reflex distance-1 (MRD-1) with blepharoptosis surgery. ΔMRD-1 equal to 2.5 mm was the cutoff point to determine whether superficial punctate keratopathy improve or unchanged group from the deterioration group, and its area under the curve was 0.6605.

**Fig. 4:**
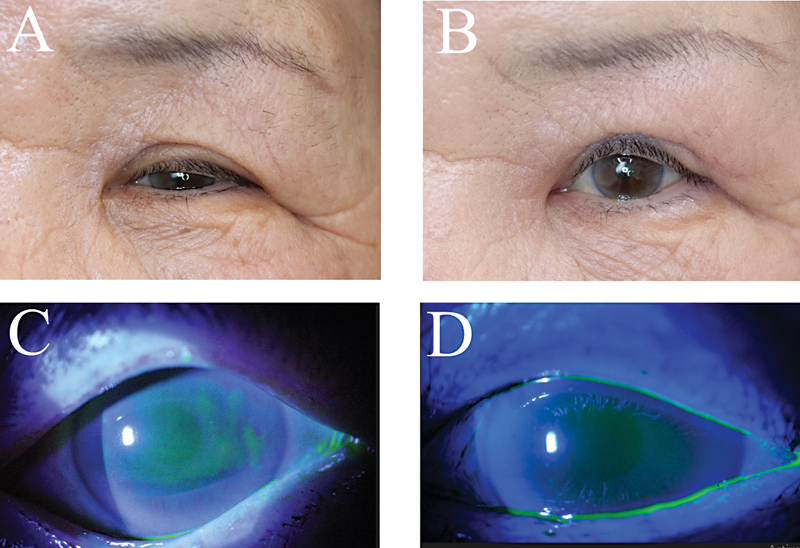
Case 1 was a 75-year-old Japanese woman. The preoperative margin reflex distance-1 (MRD-1) was 1 mm, and the postoperative MRD-1 increased to 3.0 mm, an increase of 2.0 mm ( **A, B** ). Superficial punctate keratopathy (SPK) was not observed either preoperatively or postoperatively ( **C, D** ). Case 2 was a 73-year-old Japanese woman. The preoperative MRD-1 was 1 mm, and the postoperative MRD-1 increased to 3.5 mm, an increase of 2.5 mm ( **A, B** ). SPK was not observed preoperatively, but showed mild areas and density of SPK (A1D1) postoperatively ( **C, D** ).

1. *Change of MRD-1* : the median (interquartile range) MRD-1 was 1 mm (0.125–1.5) preoperatively and 2.5 mm 2 3 postoperatively, representing a significant postoperative improvement ( *p* < 0.05, Wilcoxon signed-rank test) in Fig. 2 .
2. *Evaluation by questionnaire survey* : all 10 items on the questionnaire tended to have increased scores after surgery, and significant differences were observed in 7 items (poor visibility, ocular fatigue, heavy eyelid, foreign body sensation, difficulty in focusing, headaches, and stiff shoulders ( Table 1 ).
3. *Change of SPK scale* : the mean SPK area was 0.25 ± 0.44 preoperatively and 0.39 ± 0.58 postoperatively. The mean SPK density was 0.3 2 ± 0.6 preoperatively and 0.5 ± 0.76 postoperatively, representing a significant postoperative increase ( *p* < 0.05, Wilcoxon signed-rank test) in Table 2 .
4. *Cut-off of MRD-1 amount of change graded by SPK* : ΔMRD-1 equal to 2.5 mm was the cutoff point to separate the SPK improvement/unchanged group from the deterioration group, and its area under the curve was 0.6605 ( Fig. 3 ).
5. *Degree of exacerbation of SPK based on change in MRD-1* : SPK area and density significantly deteriorated in the group with more than or equal to ΔMRD 2.5 mm ( Table 3 ).
6. *Representative cases* : case 1 was a 75-year-old Japanese woman. The preoperative MRD-1 was 1 mm, and the postoperative MRD-1 increased to 3.0 mm, an increase of 2.0 mm. SPK was not observed either preoperatively or postoperatively ( Fig. 4A–D ).

**Fig. 5:**
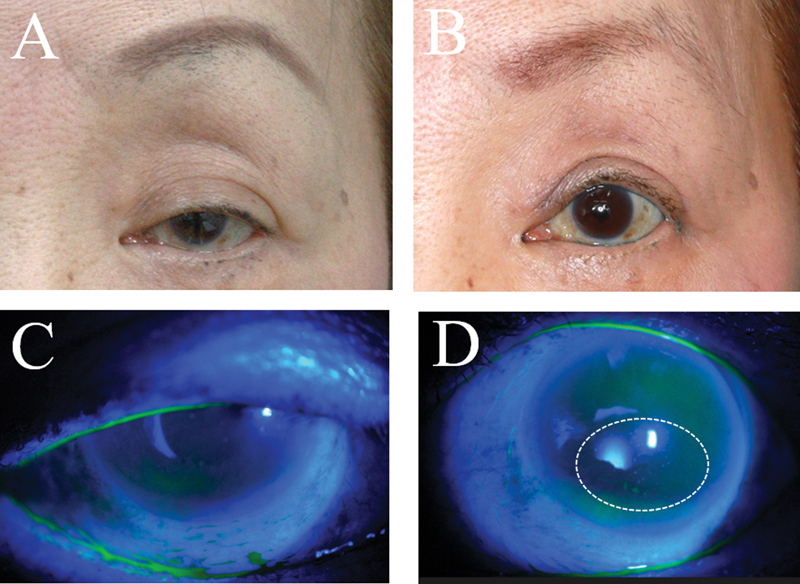
Case 2 was a 73-year-old Japanese woman. The preoperative MRD-1 was 1 mm, and the postoperative MRD-1 increased to 3.5 mm, an increase of 2.5 mm ( **A, B** ). SPK was not observed preoperatively, but showed mild areas and density of SPK (A1D1) postoperatively ( **C, D** ).

Case 2 was a 73-year-old Japanese woman. The preoperative MRD-1 was 1 mm, and the postoperative MRD-1 increased to 3.5 mm, an increase of 2.5 mm. SPK was not observed preoperatively, but showed mild areas and density of SPK (A1D1) postoperatively ( Fig. 5A–D ).

**Table 1:** All ten items on the questionnaire tended to have increased scores after surgery, and significant differences were observed in seven items

Symptoms	Pre	Post
Poor visibility	2 (1–4)	5 a (4–5)
Ocular fatigue	2 (2–3)	4 a (3–4)
Heavy eyelid	2 (1–3)	4 a (3–5)
Painful or sore eyes	4 (4–5)	5 (3–5)
Dry sensation in eyes	3.5 (2–4)	4 (3–5)
Foreign body sensation	3 (2–4)	4 a (3–5)
Hard to focus	4 (2–5)	5 a (4–5)
Double vision	4 (3–5)	5 (4–5)
Headaches	4 (3–5)	5 a (4–5)
Stiff shoulders	3 (2–4)	4 a (3–5)

Abbreviations: Pre, preoperative; Post, postoperative at 3 months.

a *p* < 0.05.

**Table 2:** The mean SPK area was 0.25 ± 0.44 preoperatively and 0.39 ± 0.58 postoperatively. The mean SPK density was 0.3 2 ± 0.6 preoperatively and 0.5 ± 0.76 postoperatively, representing a significant postoperative increase

	Pre	Post	*p* -Value
SPK area	0.25 ± 0.44	0.39 ± 0.58	0.0832
SPK density	0.32 ± 0.6	0.5 ± 0.76	0.0308

Abbreviations: Pre, preoperative; Post, postoperative at 3 months; SPK, superficial punctate keratopathy.

A comparison of the pre- and postoperative parameters. Values are presented as mean ± standard deviation. *p* -Value determined Wilcoxon signed-rank test.

**Table 3:** SPK area and density significantly deteriorated in the group with ≥ ∆MRD 2.5 mm

ΔMRD-1	≥2.5 mm	≤ 2.0 mm	*p* -Value
ΔSPK area	0.38 (0.5)	0 (0.47)	0.0196
ΔSPK density	0.43 (0.51)	0.04 (0.51)	0.0183

Abbreviations: MRD-1, margin reflex distance-1; SPK, superficial punctate keratopathy.

The amount of change in SPK A and D was examined by dividing into groups with changes in MRD-1 of 2.5 mm or more and less than 2.5 mm.

## Discussion

SPK is a petechial and multiple corneal epithelial defect. 18 It may indicate conditions that are more severe than SPK, such as corneal erosion and persistent epithelial defect. As SPK worsens, symptoms and discomfort related to the eyes are more likely to appear. 19 To our knowledge, this is the first study to report the pre- and postoperative changes in SPK in patients with BP in detail. SPK is known to mainly occur below the cornea after BP surgery. Asamura et al 6 reported that postoperative MRD-1 should be less than 3 mm because of the possible appearance of SPK due to BP surgery. In this study, SPK area and density scores were found to increase when the MRD-1 increase was more than 2.5 mm with surgery. In other words, BP surgery was found to be a safe and effective way to keep the increase in MRD-1 within 2.0 mm, especially in elderly patients.

Some surgeons tend to believe that the more MRD-1 increases, the greater the expansion of the field of vision and the more satisfied the patient will be with the surgery. The upper eyelid opens and closes along the curvature of the eyeball, and the eyelid moves flexibly. Surgeons sometimes detect a loss of this flexibility after BP surgery, and the dry eye symptoms can be exacerbated due to decreased stability of the tear film and increased friction forces that exist during blinking. The condition for SPK worsens, resulting in patient complaints. 3 6 20 21 BP surgery in elderly people must therefore be considered to be a very delicate procedure. There have been no previous reports of a scale evaluation method for SPK by surgery. SPK scale classification is based on area and density. A scale evaluation method for SPK reported by Miyata et al 17 was used in this study, and the area and density of SPK were evaluated in the groups with postoperative MRD-1 of more than 2.5 mm or less than 2 mm ( Table 3 ). Increase in MRD-1 more than 2.5 mm is suggested to be associated with the risk of worsening condition for SPK ( Fig. 3 and Table 3 ).

According to Japanese guidelines, the surgical indication for BP is MRD-1 less than or equal to 2 mm. When the MRD value is 2 mm, the upper field of view corresponds to 35 degrees, 1 mm corresponds to 30 degrees, and 0 mm corresponds to 20 degrees. The upper field of view required in daily life is set to 30 degrees, and related inconvenience tends to begin to be reported when MRD-1 value is around 1 mm. 10 22 Guillon et al 23 also reported that the pupils become smaller as an individual gets older, but stated that the diameter of the pupils at 55 years and older is approximately 3.5 mm. According to Telek et al, 24 the pupils become smaller as an individual gets older, and the diameter of the pupils at 60 years and older is approximately 3 mm. Elsewhere, Murchison et al 25 reported the average MRD-1 in a cohort of Caucasian individuals with an average age as 2.7 mm. Additionally, the function of the meibomian glands begins to decline around age 50, and approximately 70% of Japanese over age 60 develop dry eye symptoms. 26 27 Based on these reports, a position where the upper eyelid margin does not interfere with the pupil, as in the patients in this study, a postoperative MRD-1 of around 2.5 mm seems appropriate for BP surgery in elderly patients. The primary surgical purpose of BP surgery is to widen the visual field by upward displacement of the upper eyelid margin ( Fig. 1 ).

Unlike other fields of medicine that use morbidity and mortality rates as the basis for evaluation, improvement in various eye-related symptoms, such as those seen in these cases, is an important factor in the field of plastic surgery. Previous reports concluded that BP surgery provides significant improvement in vision, peripheral vision, and quality-of-life activities. 9 28 In this survey, there was significant improvement in many symptoms: poor visibility, ocular fatigue, heavy eyelid, foreign body sensation, difficulty in focusing, and also accompanying symptoms of headaches and stiff shoulders, concomitant symptoms of conditions requiring BP surgery. 9 28 In other words, BP surgery has resulted in marked improvement in many aspects of patients' subjective dry eye symptoms and health-related quality of life.

This evaluation method for SPK should also be helpful in various situations, such as when performing evaluation of the ocular surface for various types of oculoplastic surgery (e.g., entropion), when describing the time course of changes in SPK area and density. A major limitation of this study is that it only evaluated those who received postoperative SPK at 3 months, although it is possible that they may improve after 1 year. 29 30 31 BP surgery in elderly people is expected to continue to increase in the current hyper-aged society. Despite the benefits, surgeons must nonetheless be aware that BP surgery is a delicate procedure, especially in elderly people and is found to be a safe and effective way to maintain the increase in MRD-1 within 2.0 mm.

## References

[1] Hsu A K Jen A Estimation of skin removal in aging Asian blepharoplasty Laryngoscope 2012 122 04 762 766 22344689 10.1002/lary.22444

[2] Huijing M A van der Palen J van der Lei B The effect of upper eyelid blepharoplasty on eyebrow position J Plast Reconstr Aesthet Surg 2014 67 09 1242 1247 24939828 10.1016/j.bjps.2014.05.022

[3] Kim H H De Paiva C S Yen M T Effects of upper eyelid blepharoplasty on ocular surface sensation and tear production Can J Ophthalmol 2007 42 05 739 742 17823642 10.3129/i07-141

[4] Noma K Takahashi Y Leibovitch I Kakizaki H Transcutaneous blepharoptosis surgery: simultaneous advancement of the levator aponeurosis and Müller's muscle (levator resection) Open Ophthalmol J 2010 4 71 75 21293731 10.2174/1874364101004010071 PMC3032226

[5] Uemura K Shirakawa U Okuda K Essence of blepharoptosis surgery does raise the eyelids, is not just cosmetic improvement: especially in the elderly patient J Wakayama Med Soc 2021 73 9 14

[6] Asamura S Wada Y Tanaka S Saika S Study to the effect of involutional blepharoptosis surgery using objective and subjective parameters Arch Plast Surg 2022 49 04 473 478 35919549 10.1055/s-0042-1751101 PMC9340170

[7] Parsa F D Wolff D R Parsa N N Elahi aE E Upper eyelid ptosis repair after cataract extraction and the importance of Hering's test Plast Reconstr Surg 2001 108 06 1527 1536, discussion 1537–1538 11711923 10.1097/00006534-200111000-00014

[8] Finsterer J Ptosis: causes, presentation, and management Aesthetic Plast Surg 2003 27 03 193 204 12925861 10.1007/s00266-003-0127-5

[9] Bahceci Simsek I Association of upper eyelid ptosis repair and blepharoplasty with headache-related quality of life JAMA Facial Plast Surg 2017 19 04 293 297 28253391 10.1001/jamafacial.2016.2120 PMC5815105

[10] Cahill K V Bradley E A Meyer D R Functional indications for upper eyelid ptosis and blepharoplasty surgery: a report by the American Academy of Ophthalmology Ophthalmology 2011 118 12 2510 2517 22019388 10.1016/j.ophtha.2011.09.029

[11] Federici T J Meyer D R Lininger L L Correlation of the vision-related functional impairment associated with blepharoptosis and the impact of blepharoptosis surgery Ophthalmology 1999 106 09 1705 1712 10485538 10.1016/S0161-6420(99)90354-8

[12] Moesen I van den Bosch W Wubbels R Paridaens D Is dry eye associated with acquired aponeurogenic blepharoptosis? Orbit 2014 33 03 173 177 24660884 10.3109/01676830.2014.881889

[13] Bellinvia G Klinger F Maione L Bellinvia P Upper lid blepharoplasty, eyebrow ptosis, and lateral hooding Aesthet Surg J 2013 33 01 24 30 23277617 10.1177/1090820X12468751

[14] Lee J W Baker S R Esthetic enhancements in upper blepharoplasty Clin Plast Surg 2013 40 01 139 146 23186764 10.1016/j.cps.2012.08.008

[15] Har-Shai Y Hirshowitz B Extended upper blepharoplasty for lateral hooding of the upper eyelid using a scalpel-shaped excision: a 13-year experience Plast Reconstr Surg 2004 113 03 1028 1035, discussion 1036 15108902 10.1097/01.prs.0000105652.09882.b8

[16] Pereira L S Hwang T N Kersten R C Ray K McCulley T J Levator superioris muscle function in involutional blepharoptosis Am J Ophthalmol 2008 145 06 1095 1098 18374300 10.1016/j.ajo.2008.02.002

[17] Miyata K Amano S Sawa M Nishida T A novel grading method for superficial punctate keratopathy magnitude and its correlation with corneal epithelial permeability Arch Ophthalmol 2003 121 11 1537 1539 14609908 10.1001/archopht.121.11.1537

[18] Nishida T Chikama T Sawa M Miyata K Matsui T Shigeta K Differential contributions of impaired corneal sensitivity and reduced tear secretion to corneal epithelial disorders Jpn J Ophthalmol 2012 56 01 20 25 22071673 10.1007/s10384-011-0105-4

[19] Nishida T Tanaka T Extracellular matrix and growth factors in corneal wound healing Curr Opin Ophthalmol 1996 7 04 2 11 10.1097/00055735-199608000-00002 10163634

[20] Watanabe A Kakizaki H Selva D Short-term changes in tear volume after blepharoptosis repair Cornea 2014 33 01 14 17 24212766 10.1097/ICO.0000000000000010

[21] Watanabe A Selva D Kakizaki H Long-term tear volume changes after blepharoptosis surgery and blepharoplasty Invest Ophthalmol Vis Sci 2014 56 01 54 58 25425312 10.1167/iovs.14-15632

[22] Averbuch-Heller L Leigh R J Mermelstein V Zagalsky L Streifler J Y Ptosis in patients with hemispheric strokes Neurology 2002 58 04 620 624 11865142 10.1212/wnl.58.4.620

[23] Guillon M Dumbleton K Theodoratos P Gobbe M Wooley C B Moody K The effects of age, refractive status, and luminance on pupil size Optom Vis Sci 2016 93 09 1093 1100 27232893 10.1097/OPX.0000000000000893 PMC5006796

[24] Telek H H Erdol H Turk A The effects of age on pupil diameter at different light amplitudes Beyoglu Eye J 2018 3 02 80 85

[25] Murchison A P Sires B A Jian-Amadi A Margin reflex distance in different ethnic groups Arch Facial Plast Surg 2009 11 05 303 305 19797091 10.1001/archfacial.2009.9

[26] Amano S Inoue K Estimation of prevalence of meibomian gland dysfunction in Japan Cornea 2017 36 06 684 688 28410360 10.1097/ICO.0000000000001208

[27] Uchino M Dogru M Yagi Y The features of dry eye disease in a Japanese elderly population Optom Vis Sci 2006 83 11 797 802 17106406 10.1097/01.opx.0000232814.39651.fa

[28] Mokhtarzadeh A McClelland C Lee M S Smith S Harrison A R The Bleph and the Brain: the effect of upper eyelid surgery on chronic headaches Ophthal Plast Reconstr Surg 2017 33 03 178 181 10.1097/IOP.0000000000000686 27015241

[29] de Figueiredo A R Blepharoptosis Semin Ophthalmol 2010 25 03 39 51 20590412 10.3109/08820538.2010.496695

[30] Massry G G Ptosis repair for the cosmetic surgeon Facial Plast Surg Clin North Am 2005 13 04 533 539, vivi. 16253840 10.1016/j.fsc.2005.06.005

[31] Frueh B R Musch D C McDonald H M Efficacy and efficiency of a small-incision, minimal dissection procedure versus a traditional approach for correcting aponeurotic ptosis Ophthalmology 2004 111 12 2158 2163 15582068 10.1016/j.ophtha.2004.07.019

